# A Modified LeNet CNN for Breast Cancer Diagnosis in Ultrasound Images

**DOI:** 10.3390/diagnostics13172746

**Published:** 2023-08-24

**Authors:** Sathiyabhama Balasubramaniam, Yuvarajan Velmurugan, Dhayanithi Jaganathan, Seshathiri Dhanasekaran

**Affiliations:** 1 Computer Science and Engineering, Sona College of Technology, Salem 636005, India; yuvi.cn@gmail.com (Y.V.); dhaya.j@sonatech.ac.in (D.J.); 2 Department of Computer Science, UiT The Arctic University of Norway, 9037 Tromso, Norway

**Keywords:** deep learning, breast cancer, convolutional neural networks, LeNet, medical image processing, batch normalization

## Abstract

Convolutional neural networks (CNNs) have been extensively utilized in medical image processing to automatically extract meaningful features and classify various medical conditions, enabling faster and more accurate diagnoses. In this paper, LeNet, a classic CNN architecture, has been successfully applied to breast cancer data analysis. It demonstrates its ability to extract discriminative features and classify malignant and benign tumors with high accuracy, thereby supporting early detection and diagnosis of breast cancer. LeNet with corrected Rectified Linear Unit (ReLU), a modification of the traditional ReLU activation function, has been found to improve the performance of LeNet in breast cancer data analysis tasks via addressing the “dying ReLU” problem and enhancing the discriminative power of the extracted features. This has led to more accurate, reliable breast cancer detection and diagnosis and improved patient outcomes. Batch normalization improves the performance and training stability of small and shallow CNN architecture like LeNet. It helps to mitigate the effects of internal covariate shift, which refers to the change in the distribution of network activations during training. This classifier will lessen the overfitting problem and reduce the running time. The designed classifier is evaluated against the benchmarking deep learning models, proving that this has produced a higher recognition rate. The accuracy of the breast image recognition rate is 89.91%. This model will achieve better performance in segmentation, feature extraction, classification, and breast cancer tumor detection.

## 1. Introduction

Breast cancer is one of the most common causes of death in women worldwide. Early detection saves the life of many women. Healthcare practitioners extensively use mammograms for screening breast cancer. Breast ultrasound and diagnostic mammogram are the two imaging tests that are used to evaluate breast tissue for abnormalities or signs of breast cancer. While both are imaging studies of the breast, they differ in how they use sound and X-rays to obtain images and the information they provide [1].

A diagnostic mammogram is a low-dose X-ray that provides detailed images of the breast tissue. It can detect calcifications, masses, and abnormalities indicating cancer or other conditions. Diagnostic mammograms are typically recommended for women with a breast lump, nipple discharge, and women at risk of breast cancer [2,3] due to family history or other related diseases.

Hubbard et al. [4] have presented a cohort study report which discusses breast cancer risk assessment, screening guidelines in average-risk women, and certain controversies surrounding breast cancer screening. It presents recommendations for using a framework of shared decision-making to assist women in balancing their values regarding the benefits and harms of screening at various ages and intervals to make personal screening choices within a range of reasonable options. It includes recommendations for women at elevated risk and discusses new technologies like tomosynthesis [4].

A breast ultrasound normally uses a high-pitched sound to create an image of the breast. It can provide more detailed information about the structure of a breast mass, such as whether it is a fluid-filled cyst or a solid lump. Ultrasound is often used as a follow-up test to mammography as a screening [3,5].

Early diagnosis and accurate diagnosis are important for effective treatment. Computer-aided diagnosis (CAD) is a method that uses machine learning and deep learning algorithms to help doctors diagnose cancer [6]. CAD can be used at all stages of cancer screening, including mammography, ultrasound, and magnetic resonance imaging (MRI). CAD systems analyze images and provide radiologists with visualization and diagnostic information, helping them identify abnormalities that might be overlooked. CAD can detect calcifications, masses, and other breast cancer symptoms. The system then generates a report of the findings and recommends action. The stages in the table CAD system are preprocessing, segmentation, feature extraction, and classification. Additionally, the segmentation and identification of stages are difficult for researchers to diagnose breast cancer accurately. Therefore, advanced tools and techniques are required to accurately diagnose and classify breast cancer cases [7].

CAD is used to support radiologists and healthcare practitioners to make accurate diagnoses. In addition, it helps them to suggest and recommend suitable prescriptions and treatments for care. Researchers report the effectiveness of CAD in cancer diagnosis and find it to have better sensitivity and specificity. In addition, CAD can significantly reduce the time required for radiologists and healthcare practitioners to interpret images [8].

CAD is also being used to predict breast cancer risk. By analyzing factors such as age, family history, and breast density, CAD can generate a personalized risk assessment that can help guide screening and treatment decisions. CAD is a valuable tool in breast cancer analysis that improves breast cancer detection accuracy, reduces unnecessary biopsies, and treatments, and provides personalized risk assessments [5]. As technology advances, CAD will be increasingly important in breast cancer diagnosis and management.

By leveraging the ability of deep learning networks to learn intricate representations, these networks can achieve high accuracy rates in breast cancer detection, aiding in early diagnosis and reducing false positives and false negatives. Deep learning has succeeded highly in medical image processing, Computer Vision (CV), natural language processing, and video/speech recognition. Deep learning networks can assist in predicting patient outcomes and tailoring treatment plans based on individual characteristics. These networks can identify different breast cancer subtypes and stages via analyzing large-scale clinical and test data. This information can guide oncologists in selecting appropriate therapies and optimizing treatment strategies. Breast cancer screening and diagnosis can be time-consuming and labor-intensive for radiologists and pathologists. Deep learning networks can automate various stages of the diagnostic workflow, such as image analysis, lesion segmentation, and classification, leading to increased efficiency, reduced human error, and potentially faster turnaround times. Breast cancer analytics using deep learning networks enhance accuracy, efficiency, and provide personalized care in breast cancer detection, diagnosis, and treatment, ultimately improving patient outcomes and saving lives [8].

Arevalo, J. et al. [9] have proposed a hybrid representation learning framework for breast cancer diagnosis in mammography that integrated convolutional neural networks (CNNs) to learn discriminative features automatically. They have applied a biopsy-proven benchmarking dataset (344 breast cancer patients) containing 736 film mammography (mediolateral oblique and craniocaudal) views, representing manually segmented lesions associated with masses: 426 benign and 310 malignant lesions. The developed method comprises two main stages: (i) preprocessing to enhance image details and (ii) supervised training for learning both the features and the breast imaging lesions classifier in a supervised way instead of designing particular descriptors to explain the content of mammography images.

Rampun, A. et al. [10] have explored ensemble deep learning for breast mass classification in mammograms. The authors have modified the Alex Net to adapt it to the breast mass classification problem. A model selection is performed to select the best three results based on the highest validation accuracies. Then, the prediction is made based on the average probability of the models. The results show that accuracy from individual models ranges between 75% and 77%. However, the ensemble networks provide 80% accuracy and area under the curve.

Choosing appropriate algorithms for image enhancement, segmentation, feature extraction, feature selection, and prediction is important for cancer diagnosis. It is an important research area where many researchers have analyzed these datasets using deep learning algorithms [11,12]. Several deep learning algorithms can be applied to breast cancer data analytics. These algorithms leverage the power of neural networks to analyze complex patterns in the data and make predictions. Convolutional neural networks (CNN) are commonly used in deep learning algorithms for breast cancer data analytics. The specific choice of algorithm depends on the nature of the data, the research or clinical question, and the available computational resources [13]. The features can be selected after feature selection and then classified into normal, benign, and malignant classes. By leveraging the hierarchical nature of CNNs, the complex patterns and structures can be analyzed within medical images, aiding in tasks such as tumor detection, disease classification, and image segmentation [13].

The primary objectives of breast cancer classification are:

- To accurately distinguish between malignant (cancerous) and benign (non-cancerous) breast lesions.
- To improve treatment outcomes and survival rates, using the LeNet model to assist in identifying subtle abnormalities in breast images that may indicate the presence of cancer at an early stage.
- To reduce false positives and false negatives, the LeNet model can be trained to strike a balance between sensitivity (detecting true positive cases) and specificity (avoiding false positives) and enhance the accuracy of breast cancer classification.
- To provide prognostic information, predict the likelihood of disease progression and patient survival, and to help assess the risk of recurrence and guide decisions regarding post-treatment surveillance and follow-up care.
- To recommend tailoring treatment approaches to individual patients. By classifying tumors based on specific molecular markers or genetic profiles, personalized treatment plans can be developed and therapeutic outcomes can be optimized.
- It is important to note that the modified LeNet can serve as a foundation for breast cancer classification. The architecture offers increased model depth, enabling the learning of more complex features, and potentially achieving higher classification accuracy.

## 2. Literature Review

Breast cancer is one of the most common diseases among women, and early detection is a crucial step to save the lives of millions of women. Due to the recent developments in healthcare, breast cancer patients can be diagnosed at an early stage. More importantly, conventional methods of analyzing breast cancer images suffer from high false detection rates. Different breast cancer imaging modalities are used to extract and analyze the key features affecting the diagnosis and treatment of breast cancer. These imaging modalities can be divided into subgroups such as mammograms, ultrasound, magnetic resonance imaging, histopathological images, or any combination [14]. Radiologists or pathologists analyze images produced by these methods manually, which leads to an increase in the risk of wrong decisions for cancer detection. Thus, the utilization of new automatic methods to analyze all kinds of breast screening images to assist radiologists in interpreting images is required.

Artificial intelligence (AI) has recently been widely utilized to automatically improve the early detection and treatment of different types of cancer, specifically breast cancer, thereby enhancing the survival chance of patients. Advances in AI algorithms, such as deep learning, and the availability of datasets obtained from various imaging modalities have opened an opportunity to surpass the limitations of current breast cancer analysis methods. In this article, breast cancer imaging modalities, and their strengths and limitations are discussed. Then, the most recent studies that have employed AI in breast cancer detection and various breast imaging modalities have been summarized. In addition to this, the paper presents the datasets on the breast-cancer imaging modalities, which are important in developing AI-based algorithms and through offering training of deep learning models [14].

Recently, there has been a great demand for the classification of breast cancer using ultrasound images and mammography [15]. Mammograms, ultrasound scans, and MRI imaging are specific imaging modalities in breast cancer screening and diagnosis that radiologists and oncologists majorly use. CAD can further support healthcare practitioners in detecting and diagnosing breast cancer. Early detection of pectoral muscles becomes necessary in reducing the ambiguities which could provide movements to the upper limbs or ribs. Their recognition is essential in enhancing the diagnostic performance of breast cancer detection.

A deep convolutional neural network is used to classify the 1.2 million high-resolution images in the ImageNet [16]. On the test data, top-1 and top-5 error rates are 37.5% and 17.0%, respectively. In the neural network, five convolutional layers are followed by max-pooling layers, and three fully connected layers with a final 1000-way SoftMax. To make training faster, they used GPU implementation of the convolution operation. To reduce overfitting, the authors used a “dropout” regularization method that proved very effective. They are the winners of top-5 test error rate of 15.3% in the ILSVRC-2012 competition, compared to 26.2% achieved by the second-best entry [16].

The authors [17] have designed a deep feature-based framework for breast mass classification using CNN and a decision mechanism. The combined intensity information and deep features have been automatically extracted using the trained CNN. Then, classifiers are used to predict classes of test images. Their method is applied to the DDSM dataset and achieved high accuracy and classification performance.

Vidhushavarshini et al. [18] have designed a simple machine learning classifier for analyzing thyroid datasets. This model used the patients’ history to predict the disease via creating the knowledge prediction model, easing the medical practitioner’s job with better accuracy.

Zahoor et al. [19] have designed a CAD-based classification model to prevent the disease as well as to reduce the risk of breast cancer in women. The CAD system’s accuracy was improved via reducing the false-positive rates. The modified entropy whale optimization algorithm (MEWOA) was used to optimize the features. The fine-tuned MobilenetV2 and NASNet Mobile are applied for simulation. Then, the machine learning classifiers are applied to classify breast cancer images using the optimized deep features. Three publicly available datasets are used to extract the features and perform the classification: INbreast, MIAS, and CBIS-DDSM. Their classification accuracies are 99.7%, 99.8%, and 93.8% and their performance is reasonably good compared to other approaches [19].

Deep learning networks in breast cancer analytics have emerged as a promising approach to improve early detection, diagnosis, and personalized treatment. Deep learning networks excel in analyzing such vast amounts of data, allowing for improved detection and classification of breast cancer. Deep learning networks can automatically learn complex patterns and features from breast images without explicit feature engineering. Breast cancer detection and diagnosis often require the analysis of subtle, and localized abnormalities, which can be challenging for traditional machine learning methods [6].

The researchers have applied appropriate pre-processing steps and have improved the input images in contrast to improving the target detection accuracy [13]. For example, Sadad et al. [20] have developed a CAD model to diagnose cancer and to reconstruct the images using Hilbert Transform (H.T.) for better visualization. Next, they have segmented the tumor using a marker-controlled basin transformation. In the next step, shape and texture features were extracted and classified using a hybrid k-nearest neighbors algorithm (KNN) and cluster decision tree model. Badawi et al. [21] have used fuzzy logic in the first step and defined the region of interest using a semantic segmentation method. Then, they have used eight pre-trained models for accurate tumor classification. The authors have used image processing, machine learning, and deep learning to diagnose cancer [21]. They have used reliable methods to detect breast cancer early to save women’s lives: microcalcifications and the malignant cells CAD system for breast cancer detection. The test results show that the CAD system can detect breast cancer initially. Segmentation and distribution of stages in this model are unsuitable for breast cancer diagnosis.

Zahoor et al. [22] have presented image processing, machine learning, and deep learning methods to diagnose breast cancer. The authors have highlighted better choices and reliable methods to diagnose breast cancer in the initial stages to save women’s lives . Additionally, several CAD methodologies were discussed to detect breast masses, microcalcifications, and malignant cells [22]. Lévy, D. and Jain [23] have designed a hybrid model combining CNN and transfer learning to classify breast masses in mammograms as benign or malignant. The authors added pre-processing and data augmentation to overcome the inadequate training data issues. They achieved a better performance on the DDSM dataset [23].

Ting et al. [24] have presented an improved CNN for breast cancer classification to assist the medical practitioners. This improved CNN model classifies the breast lesions into benign, malignant, and normal with 90.5% accuracy, 89.47% sensitivity, 90.71% specificity, and 0.901 receiver operating characteristics [24]. Vidhushavarshini S et al. [25] have proposed a hybrid optimization algorithm-based feature selection design for thyroid disease classification with rough type-2 fuzzy support vector machine. This work combined the firefly algorithm (FA) and the butterfly optimization algorithm (BOA) namely hybrid firefly butterfly optimization-rough type-2 fuzzy support vector machine (HFBO-RT2FSVM) to select the top-n relevant features. HFBO-RT2FSVM is evaluated with several key metrics such as specificity, accuracy, and sensitivity. We compare our approach with well-known benchmark methods such as improved gray wolf optimization linear support vector machine (IGWO Linear SVM) and mixed-kernel support vector machine (MKSVM) methods. The HFBO-RT2FSVM technique improved the classification accuracy and thereby precision in thyroid disease identification. The HFBO-RT2FSVM model attained an accuracy of 99.28%, having specificity and sensitivity of 98% and 99.2%, respectively.

Dense breast tissue is an independent risk factor for breast cancer and it lowers the sensitivity of screening mammography [26]. Hence, medical practitioners rely on supplemental screening like ultrasound or MRI to improve breast cancer detection rate. Supplemental screening is also influenced by a history of breast biopsy and family history of breast cancer, race, age, socioeconomic status, density category, and physician’s specialty and region [26].

Feature extraction is a very important step when mammography image analysis is addressed using learning-based approaches [27]. Automated feature extraction is used to represent the content of images with CNN, and the classifier is evaluated to learn features from mammography mass lesions. Empirical results prove that this approach is effective in identifying the target, and that its classification accuracy ranges from 79.9% to 86% in terms of area under the ROC curve [27].

The Chan–Vese level set method [28] is used to extract the initial contour of mammograms, and a deep learning CNN (DL-CNN) is used to learn the features of mammary-specific mass and microcalcification clusters. A fully complex-valued relaxation network is used in the last stage of DL-CNN network to increase the classification accuracy. Experiments are conducted using the standard benchmarking breast cancer datasets MIAS and the Breast Cancer Digital Repository (BCDR). The results show that the proposed method has significantly improved the performance over the traditional methods. Performance measures such as accuracy, sensitivity, specificity, AUC achieved are 99%, 0.9875, 1.0 and 0.9815, respectively, and the effective classification of the mammogram images has been as normal, benign, or malignant and its subclasses [28].

Chai et al. [29] have provided a review of recent achievements in terms of techniques and applications in CV methods. The authors have identified the emerging techniques and have investigated their applications in the scenarios, including recognition, visual tracking, semantic segmentation, and image restoration. They have also discussed the future research directions, prospective growth, and impact of these technologies in various domains. The summarization, knowledge accumulation, and creation would benefit researchers in these domains and participators in the CV industries [29].

N. Borah et al. [30] have applied automation based on ViT compared to the existing systems; the effectiveness of the proposed model is in relation to mammogram images (IN breast database). Real-time performance provides 96.48% accuracy and requires little training time to analyze medical images. The suggested model is built using a graphical user interface (GUI), which might help medical professionals make wiser judgments and identify B.C. more quickly [30].

Many hybrid optimization algorithms are trapped in local optima and have slow convergence speeds, which reduces the classification accuracy. To resolve these issues, a hybrid optimization algorithm that combines the grasshopper optimization algorithm and the crow search algorithm for feature selection and classification of the breast mass with multilayer perceptron has been developed [31]. The simulation is performed using MATLAB 2019a. The model’s efficacy is compared with other related optimization algorithms, and the results are better to a reasonable degree compared to the other models in terms of classification accuracy (97.1%), sensitivity (98%), and specificity (95.4%) for the MIAS dataset [31].

Manikandan et al. [32] have studied clinical, epidemiology, and end outcome datasets to distinguish between cancer cases and deaths. This study presents a machine learning-based approach to classify SEER breast cancer data. In addition to this, SEER breast cancer data have been selected for analysis using a two-step selection method based on baseline differences and values. After selecting the features, Ada, XG, gradient, naive Bayes, and decision tree supervised and ensemble learning techniques are used to classify the breast cancer dataset. Decision trees have the highest accuracy (98%) for train–test split and cross-validation [33].

Arikidis, N. et al. [11] have experimented with a two-stage semiautomated segmentation method of microcalcification (MC) clusters. In the first stage, efficient segmentation of most of the particles of a MC cluster, and shape refinement of selected individual MCs is performed in the second stage. The effect of the proposed segmentation method on MC cluster characterization accuracy was evaluated in a case sample of 162 pleomorphic MC clusters (72 malignant and 90 benign). Ten MC cluster features, targeted to capture morphologic properties of individual MCs in a cluster (area, major length, perimeter, compactness, and spread), were extracted and a correlation-based feature selection method yielded a feature subset to feed in a support vector machine classifier. Classification performance of the MC cluster features was estimated by means of the area under receiver operating characteristic curve utilizing tenfold cross-validation methodology. Interobserver and intraobserver segmentation agreements (median and [25%, 75%] quartile range) were substantial with respect to the distance metrics HDISTcluster (2.3 [1.8, 2.9] and 2.5 [2.1, 3.2] pixels) and AMINDISTcluster (0.8 [0.6, 1.0] and 1.0 [0.8, 1.2] pixels), while moderate with respect to the AOMcluster (0.64 [0.55, 0.71] and 0.59 [0.52, 0.66]). This method outperformed (0.80 ± 0.04) statistically significantly (Mann–Whitney U-test, *p* < 0.05) the B-spline active rays segmentation method (0.69 ± 0.04), suggesting the significance of the proposed semiautomated method.

Early detection of breast cancer ensures appropriate treatment and survival, and breast cancer diagnosis is mainly based on histopathological images [33]. Therefore, CAD is used for the automatic identification and diagnosis of cancer to help doctors diagnose cancer. The authors use five ConvNets: ResNet, VGG19, VGG16, Xception, and MobileNet. Their approach supports limiting the hardware needed to complete the large-scale ConvNets training task. It outperforms other handcrafted features for histopathology images.

Various solutions based on CV have been proposed in the literature [34]. These solutions have been unsuccessful due to large video sequences that need to be processed in surveillance systems. The problem arises in the presence of multi-view cameras. Recently, the development of deep learning-based systems has shown significant success for human action recognition (HAR), even for multi-view camera systems. The authors [34] have designed a deep learning-based HAR. This classifier has multiple steps, including feature mapping, fusion, and selection. Two pre-trained models are considered for the initial feature mapping step, and the extracted deep features are fused using the Serial-based Extended approach. Then, the best features are selected using kurtosis-controlled weighted k-nearest neighborhood. Benchmark datasets, such as KTH, IXMAS, WVU, and Hollywood, are used to conduct the experiments, and achieved accuracies of 99.3%, 97.4%, 99.8%, and 99.9%, respectively [34].

CV researchers used deep learning techniques on medical images to diagnose COVID-19 patients. An automated technique was proposed using parallel fusion and optimization of deep learning models for classifying the COVID-19 data [35]. This technique starts with a contrast enhancement using a combination of top-hat and Wiener filters. Optimal features are selected using the entropy-controlled firefly optimization method. The selected features are classified using machine learning classifiers such as multiclass support vector machine (MC-SVM). Two pre-trained deep learning models AlexNet and VGG16 are used to classify the data into COVID-19 and healthy as target classes. Experiments are carried out using the Radiopaedia database and an accuracy of 98% was achieved [35].

Shervan et al. [36] have proposed a multi-layer perceptron (MLP) neural network model to analyze cervical cancer datasets. The number of hidden layer neurons is tuned and ResNet-34, and VGG-19 deep networks are used to feed the MLP. The authors [37] have modified the layers related to the classification phase in these CNN networks, and the outputs feed the MLP after passing through a flatten layer. CNNs are trained on related images using the Adam optimizer to improve performance. Herlev benchmark cervical dataset is applied to this model and achieved 99.23% and 97.65% accuracy for the two classes. The results show that this method is more accurate than the other models [36].

Tan et al. [37] have proposed a federated learning (FL) approach to overcome the conduct of experiments in central learning environments, which may breach patients’ privacy. To address these difficulties, an FL facility that extracts features from participating environments rather than a CL facility has been developed. This study’s novel contributions include (i) the application of transfer learning to extract data features from the region of interest (ROI) in an image, which aims to enable careful pre-processing and data enhancement for data training purposes; (ii) the use of a synthetic minority oversampling technique (SMOTE) to process data, which aims to classify data and improve diagnostic prediction performance for diseases more uniformly; (iii) the application of FeAvg-CNN + MobileNet in an FL framework to ensure customer privacy and personal security; and (iv) the presentation of experimental results from different deep learning, transfer learning, and FL models with balanced and imbalanced mammography datasets, which demonstrate that our solution leads to much higher classification performance than other approaches and is viable for use in AI healthcare applications.

N. S. Patil et al. [38] have designed a multi-classification framework using various magnification factors, and this dataset is used. Their model examined several transfer learning models while fine-tuning adaptation. Better results were obtained from the trials using both the DenseNet201 and DenseNet121 pre-trained models. Compared to other models, the results are improved for the multi-classification of breast cancer histopathology images [38].

R. Sanyal et al. [39] have proposed a hybrid method for classifying breast histopathology images. Its frameworks include several fine-tuned CNN architectures as supervised feature extractors and eXtreme gradient booster trees (XGBoost) as best classifiers. The model consists of multiple discriminant representations of patches and XGBoost for robust classification to optimize patches. The experimental data show that the proposed method outperforms the most advanced form.

Bagchi, A. et al. [40] have used deep learning to classify breast cancer from histopathological images. The patch classification model is used where patches are preprocessed using taint normalization, and amplification techniques. Image patches were divided into four groups benign, normal, invasive, and in situ, using machine learning-based classifiers and ensemble techniques. There are two main groups classified as cancerous and non-cancerous, and the other four groups are classified as benign, in situ, and invasive according to the model. This model used the probabilities for two classes and achieved a 97.50% classification accuracy. The model achieves classification accuracies of 97.50% and 98.6% for 4-class and 2-class image classifications, respectively, on the ICIAR BACH dataset [40].

Guleria HV et al. [15] have proposed a combination of variational auto Encoder and the Denoising Variational Auto Encoder algorithm for reconstructing the breast histopathological images. Then, they used CNN and tested this multi-classifier using Kaggle dataset to predict cancerous or non-cancerous classes. This model has produced 73% accuracy, which is much better than the traditional CNN [15].

To capture the discriminant features, a deep neural network (AHoNet) was designed to acquire in-depth regional features of cancer images. On the other hand, AHoNet uses a channel tracking strategy with low attenuation and local features located between channels. Then, power matrix normalization and second-order comparison statistics have been calculated to provide an excellent global representation of breast cancer images. The BACH breast cancer database has been extensively analyzed by AHoNet. Experimental results show that AHoNet outperforms a state-of-the-art model in this clinical application, achieving the best results at the patient level. Classification accuracy of 99.29% and 85% in the BreakHis and BACH databases, respectively, has been achieved [15].

Kumar, D. et al. [41] have designed a soft voting-based 7-CNN model. The proposed method uses a VGG 19 transfer learning model (with and without data augmentation), VGG 16 transfer learning model (without data augmentation), simple CNN with four convolution layers, simple CNN with five convolution layers (with data augmentation), and Xception. Another learning model with and without data augmentation is also used. A hematoxylin–eosin dye has been used in the experiment. The performance of each model is compared with accuracy, precision, recall, and F1 score. The main criteria is taken as the basis for the evaluation. Using the H&E dataset, the proposed method achieves an accuracy of 96.91%.

Shahidi et al. [42] have studied the ResNeXt, Dual Path Networks, SENet, and NASNet deep learning models for analyzing the breast cancer histopathology image databases. Their aim is to examine the state structure via applying the following steps: pre-processing, data augmentation, and transfer learning methods. This study has identified the most accurate models in terms of the binary, four, and eight classifications of breast cancer histopathology image databases. The experimental results show that the models like Res-NeXt, Dual Path Net, SENet, and NASNet have been identified with the most cutting-edge results for the ImageNet database.

Kathale et al. [43] have proposed a technique for identifying malignant tissue and classifying healthy and cancerous patients. The input is preprocessed using morphological operations to separate the tumor area from the mammogram and highlight the size of the raw mammogram. If the mammogram looks normal, the patient is healthy. Breast cancer patients and healthy individuals are classified using random forest classifiers, and its accuracy for various patient images is 95% [43].

Farhadi et al., 2019 [44] have designed an efficient deep transfer learning method to handle the imbalanced data problem in breast cancer data. The classifier focused on structured data and used several publicly available breast cancer datasets to generate a ‘pre-trained’ model, and transfer learned concepts to predict malignant tumors. The authors compared their results with state-of-the-art techniques for addressing the problem of imbalanced learning to handle different degrees of class imbalance; a series of experiments are performed on publicly available breast cancer data under simulated class imbalanced settings. The experimental results proved that the deep transfer learning model is used to handle imbalanced class problems effectively.

Cutting-edge CNN architectures, such as ResNet and Inception, evaluate the effectiveness of the proposed method. Experiments on various criteria, such as CIFAR-10 and CIFAR-100, have been conducted. It has been evaluated on real-world data, such as the Caltech-101 dataset containing 101 items. Finally, after going through the learning process of the image using convolution and pooling techniques, all features are extracted and put into the long tube. Hence, the extracted features are ready for classification with the help of the usual fully connected layers [45].

Pectoral muscle detection is an important task to improve the diagnostic performance of breast cancer detection. Early detection of pectoral muscles significantly reduces the ambiguity between the tumor cell and pectoral muscle. It becomes necessary to suppress the pectoral muscle to achieve appropriate segmentation. For artifacts and pectoral muscle removal, Otsu’s threshold is used to identify pectoral muscles and connected component labelling to recognize and eliminate the connected pixels outside the breast region, classes 10 and 100, respectively [46].

To help pathologists analyze cancer, CAD is introduced to automatically identify and diagnose breast cancer. The CAD system is based on deep convolution neural networks (ConvNets) in this work. Deep learning (DL) establishes an important development in artificial intelligence (AI) and has been especially successful in image processing. Nevertheless, the training of ConvNets needs a huge number of images. The transfer learning is utilized to extract features from a pre-trained network of the ImageNet challenge (ILSVRC) for further classification and used five famous ConvNets: ResNet, VGG19, VGG16, Xception, and MobileNet have been used to resolve this issue over feature extraction. This method supports limiting the hardware necessary to complete the large computation task of training ConvNets. ConvNets with ML techniques are understood to perform better than the other hand-crafted features utilized for histopathology images. From the experimental results, it is found that VGG16 combined with the SVM approach outperformed in automated histopathological image classification [47].

To enhance the diagnosis of unlabeled data, W. Sun et al. [33] have proposed a semi-supervised training program based on weighted back data, selection of the feature concept, and pre-diagnostic data collection using deep CNN model. However, many studies focused on benign and malignant differentiation, tumor localization, and detected a mass in dense breast regions and pectoral muscles, which may be difficult due to high intensities. Experimental results show that the schema only requires a few pieces of data (100 cases) for training and performs well on unlabeled data (3058 points). This deep CNN-based CAD model may help improve the radiologists’ analysis and detection of breast cancer [33].

There is a research gap in determining the ideal number of models to utilize in an ensemble, even though using LeNet CNN with ensembling has improved the accuracy of picture classification tasks [48]. Most researchers have used a fixed number of models, ranging from a few to dozens, but are yet to look into the advantages or disadvantages of changing the ensemble size. In addition, there is a lack of knowledge regarding the most effective ways to mix various models into an ensemble, particularly when the models have various architectures or hyperparameters. To increase accuracy rates in picture classification tasks, further study is required to investigate the ideal ensemble size and the best ways to mix models in an ensemble. The results of this study can demonstrate the benefits of using LeNet-CNN for clustering and provide useful suggestions on how to tune the clustering process for different datasets and applications [48].

Min-pooling layer-based LeNet model is designed for Alzheimer’s Disease (AD) classification, comparing it with 20 commonly used DNN models and related works [48]. The findings suggested that the LeNet model achieved superior performance and has significantly improved classification accuracy. The LeNet model incorporates a separate min-Pooling layer alongside the traditional MaxPooling layers, allowing preservation of important low-intensity features.

Gray wolf optimizer (GWO) with a rough set theory-based CAD system is proposed for mammogram image analysis [49]. Texture, intensity, and shape-based features are extracted from mass-segmented mammogram images. A novel dimensionality reduction algorithm is proposed based on GWO with rough set theory to derive the appropriate features from the extracted feature set. GWO is a bio-inspired optimization algorithm simulated based on hunting activities and the social hierarchy of the gray wolves. This paper uses a hybridization of GWO and rough set (GWORS) methods to find the significant features from the extracted mammogram images. To evaluate the effectiveness of the proposed GWORS, particle swarm optimizer (PSO), genetic algorithm (GA), quick reduct (QR) and relative reduct (RR) are compared. Experimental results have revealed that the proposed GWORS outperforms the other techniques regarding overall accuracy, f-measures, and receiver operating characteristic (ROC) curve [49].

Many CAD systems have been established to diagnose the disease and provide better treatment. There is still a need to improve existing CAD systems via incorporating new methods and technologies to provide more precise results. Most of the CAD models used only mammogram datasets for experimentation. CAD models in breast cancer classification have several limitations that should be considered. Some of these limitations include:

- CAD models may produce false-positive results, leading to unnecessary follow-up tests or interventions. False positives can cause patient anxiety, additional healthcare costs, and potential harm from invasive procedures. Striking a balance between sensitivity and specificity is crucial to reduce false-positive rates in CAD models.
- CAD models may have lower sensitivity in detecting certain breast lesions, such as small or subtle lesions, non-mass-like lesions, or early-stage cancers. These lesions may not exhibit clear visual cues or distinctive features that CAD models can reliably detect, leading to missed diagnoses [5,7].
- CAD models are often developed and trained on specific datasets, which may not adequately represent the population or exhibit heterogeneity in terms of demographics, imaging protocols, or lesion types. This limited generalizability can affect the performance of CAD models when applied to different populations or images.
- CAD models are often considered “black boxes” since medical professionals do not readily interpret their decision-making process. This lack of transparency and interpretability can create challenges in understanding the features or patterns driving the model’s predictions, hindering trust and acceptance from clinicians.
- Integrating CAD models into clinical practice can be challenging. Incorporating CAD systems requires workflow adjustments, radiologist training, and potential integration with existing healthcare information systems. Adoption barriers, resistance to change, and logistical constraints may hinder the effective integration of CAD models into routine clinical workflows.

Addressing these limitations and continuously refining CAD models through the proposed research maximizes their potential in breast cancer classification and improves patient outcomes. The proposed research focuses on developing advanced image analysis techniques and feature extraction methods to improve the discriminative power of CAD models. Breast cancer datasets often suffer from class imbalance, where the number of malignant samples is significantly lower than that of benign samples. Imbalanced data can lead to biased model performance and reduced sensitivity in detecting cancerous cases.

Developing techniques to address these research gaps leads to more accurate, interpretable, and clinically useful CAD models to ensure reliable and flexible classification.

1. The proposed modified LeNet, like many other deep learning models, is designed for performance and aims to classify images accurately.
2. Modified LeNet demonstrates promising performance on breast cancer ultrasound datasets; their generalizability and applicability to real-world clinical settings, evaluating their impact on clinical decision-making and patient outcome, are better than any other CAD models.

LeNet [48] is a specific and relatively simple architecture within the broader category of CNNs. LeNet typically has a shallower architecture compared to more recent CNN models, which can limit its ability to capture intricate and complex features in breast cancer images. LeNet is a simple CNN-type architecture; the authors have proposed the following modifications, which can be considered to improve its classification performance for breast cancer [48]. To increase the depth, more convolutional and fully connected layers have been added, enhancing the model’s ability to capture complex patterns in breast cancer images. This allows for the extraction of higher-level features that may contribute to better classification performance.

Batch normalization [17] is applied after each convolutional or fully connected layer to standardize the inputs and reduce internal covariate shifts. This technique helps stabilize and accelerate the training process, improving classification accuracy. Dropout regularization [22] can reduce overfitting via randomly dropping out a fraction of the neurons during training. This encourages the network to learn more robust and generalized features, enhancing the model’s ability to classify breast cancer images accurately and quickly. LeNet can be combined with multiple networks called ensemble methods to average or stack predictions from multiple LeNet models and can improve classification accuracy via leveraging diverse representations learned via different network instances. Thus, modified LeNet architectures for breast cancer classification, ultimately improves early detection, diagnosis, and treatment decisions for patients efficiently.

## 3. Proposed System

The convolutional layer in a CNN performs an operation where two functions, the input image and the convolution filter, are combined to generate a third variable. The essential components of a CNN include a convolutional layer, a pooling layer, a fully connected layer, and another convolutional layer.

### 3.1. CNN Model

**Figure 1:**
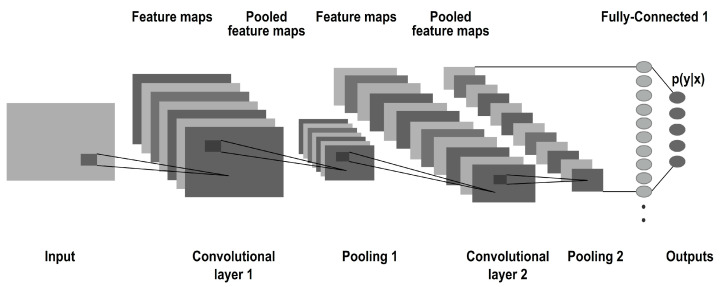
Convolutional neural network.

The image pixel values are passed through the first convolutional layer in CNN architecture which is presented in Figure 1. Each subsequent convolutional layer aims to discover relationships between the pixels and their neighboring pixels utilizing kernels to extract various features from the image [8]. CNN typically consists of a convolutional layer, which applies a set of learnable filters to the input image. These filters detect various features such as edges, textures, and patterns. The output of the convolutional layer is a feature map that highlights the presence of these features in the image. Following the convolutional layer, a common choice is to use a pooling layer, such as max pooling or average pooling. This layer reduces the spatial dimensions of the feature map while preserving the most important features. Pooling helps to downsample the data, reducing the computational complexity and extracting the most relevant information.

**Figure 2:**
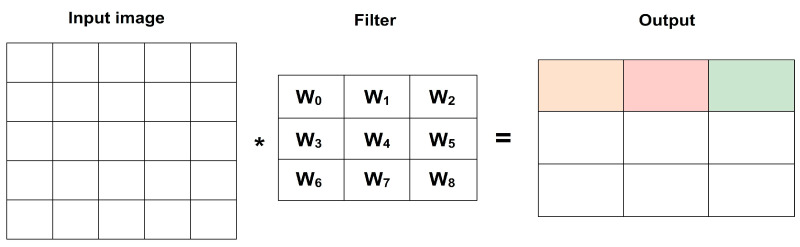
CONV layer.

After the pooling layer, additional convolutional and pooling layers can be stacked to capture increasingly complex features in the image. This hierarchical structure enables the network to learn abstract representations of the input data. To introduce non-linearity into the model and enhance its expressive power, activation functions like ReLU (Rectified Linear Unit) are applied after each convolutional layer. ReLU sets negative values to zero, preserving positive values, and introducing non-linear transformations. Towards the end of the network, fully connected layers are typically used. These layers process the extracted features and make predictions based on them. Fully connected layers connect all the neurons from the previous layer to the neurons in the current layer, enabling the network to learn complex relationships between the features and the output labels. Finally, the output layer of the CNN depends on the specific task at hand. For example, in a classification task, a SoftMax layer can be used to produce the probabilities for each class, while in a segmentation task, a convolutional layer with a pixel-wise activation function may be employed. It is important that the architecture and sequence of layers in a CNN can vary depending on the specific requirements of the task and the dataset being used. Further, the researchers often experiment with different architectures, hyperparameters, and regularization techniques to optimize performance and achieve accurate and reliable results. The result is passed on to the subsequent layers and the working of the kernel presented in Figure 2.

Figure 2 shows the CONV layer. The number of parameters in a CNN is determined by the combination of these layer sizes and depths. Each parameter corresponds to a weight value that is learned during the training process. The number of parameters in the convolutional layers is influenced by the size of the filters and the depth of the layers. More filters or larger filters lead to an increased number of parameters. The number of parameters in the fully connected layers is determined by the size of the layers, as each connection between neurons requires a weight parameter. While it is true that adding more convolutional layers can increase the number of parameters, it is not the only factor that affects parameter count. The number of parameters in a neural network architecture can be influenced by various factors, including the sizes and depths of fully connected and convolutional layers. With multiple convolutional layers, CNNs can learn increasingly abstract and complex representations of the input. Lower layers capture low-level features, while deeper layers capture high-level semantic information. Increasing the depth or size of these layers can generally lead to more parameters, but other architectural choices like pooling, strides, and padding also impact the total number of parameters. Convolutional layers apply filters to the input data, enabling them to extract meaningful features, such as edges, textures, shapes, and to generalize well. These learned features are crucial for subsequent layers to easily understand and classify the data. Convolutional layers can detect features regardless of their exact spatial location. This property makes CNNs robust to translations or shifts in the input data.

### 3.2. Fully Connected Layer

**Figure 3:**
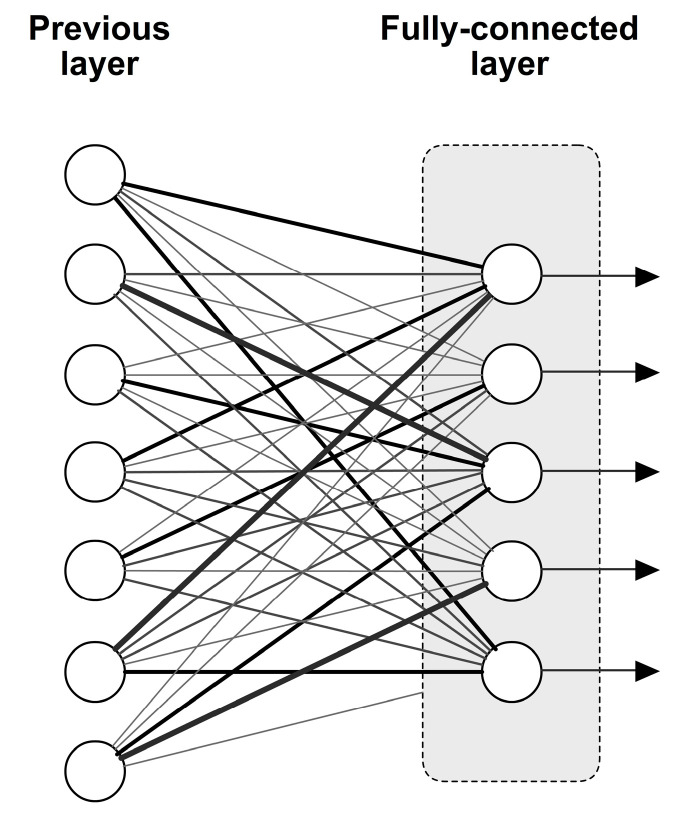
Fully connected layer.

A type of neural network layer called the fully connected layer has connections between each neuron in the upper layer which is shown in Figure 3. This layer, also known as a dense layer, is frequently employed in deep learning models to perform tasks including speech recognition, natural language processing, and image categorization [34].

A completely connected layer has a vector as input and another as output. Each neuron in the layer performs a weighted sum of the inputs and applies an activation function to produce the output. Backpropagation and gradient descent methods are used in the training process to determine the weights and biases of the fully connected layer. Every training iteration involves adjusting these weights and biases to reduce the loss of function. Fully linked layers are adaptable and robust, but they also include a lot of parameters, which, if not regularized appropriately, can result in overfitting. Deep learning models frequently employ weight decay and dropout strategies to overcome this problem [22].

## 4. LeNet Architecture

LeNet [48] is used for image classification tasks. Compared to other deep learning classifiers, LeNet has several advantages: parameter efficiency, spatial invariance, hierarchical feature learning, weight sharing, activation function, and pooling layer. A modified LeNet CNN for image classification and ensembling combines multiple models to improve accuracy. The modified LeNet architecture includes additional convolutional and pooling layers to increase model depth and capture more complex features. In addition to this, the classifier provides batch normalization [17] and continuous output to prevent overfitting and improve generalization. The limitations of current models that use LeNet for cancer diagnosis and classification are: although LeNet can be used effectively for cancer diagnosis and classification, it also has some drawbacks, which include limited capacity and flexibility, overfitting, pre-processing, and hardware requirements which are significant challenges [32]. To overcome these drawbacks, the present model aims to develop LeNet architecture with the following modifications: In the data preparation process, the Breast Ultrasound (BUS) image datasets are obtained and divided into three groups: training, validation, and testing [50]. The 60:20:20 data split refers to dividing the available dataset into three subsets, a training set (60% of the data), a validation set (20% of the data), and a test set (20% of the data). This split allows for model training on the training set, hyperparameter tuning on the validation set, and final evaluation on the test set. Hyperparameter tuning is essential to maximize the model’s performance and to find the best configuration for a specific task. It requires a careful exploration of the hyperparameter space and an understanding of the trade-offs among different choices. Through hyperparameter tuning, models can be fine-tuned to achieve optimal performance on the given dataset. Hyperparameter tuning is an important step in optimizing the performance of deep learning models. The following are the key hyperparameters that can be considered for tuning:

1. Learning rate is 0.01 which determines the step size at each iteration during training and achieves the best performance.
2. Dropout rate is 40% (0.4) which controls the amount of regularization applied to the model and prevents overfitting.
3. Batch size is 32 which determines the number of samples processed before the model’s weights are updated. This will lead to faster convergence and will result in better generalization which impacts the model’s performance.
4. Number of Hidden Units is three which can significantly impact the model’s capacity and performance and finding the optimal balance between model complexity and overfitting.
5. Number of training epochs is 10 which determines how many times the model will iterate over the entire training dataset which leads to balance between underfitting and overfitting.
6. Activation functions: The activation function used in the convolutional layers (ReLU) and the dense layer (SoftMax) can also be experimented with to determine if other activation functions yield better results for your specific problem.
7. Number of filters in the convolutional layers are 32, 64, and 128 which can be tuned to determine the complexity and capacity of the model.
8. Kernel size in the convolutional layers is 3, 5, and 4 which can be tuned to capture different spatial patterns in the input data.
9. Optimizer: The choice of optimizer can also influence the model’s performance. An optimizer is a function that modifies the attributes of the neural network, such as weights and learning rate. Common optimizers include Adam, RMSprop, and SGD with momentum. Each optimizer has different hyperparameters of its own, such as momentum or decay rates. Thus, it helps in reducing the overall loss and improving accuracy. Here, the Adam optimizer is used which is best suited for breast cancer datasets.

This approach helps in assessing the model’s performance on unseen data and avoiding overfitting. Pre-processing images are fed as input into an ML model. In the case of the LeNet model, it is necessary to pre-process the images into a format that can be effectively processed by the model. This typically entails resizing the images to a specific size, normalizing the pixel values, and potentially applying other transformations as per the model architecture’s requirements. The unique aspect of LeNet that sets it apart from many other classifier networks is its utilization of weight sharing and the choice of activation function [51]. LeNet, a classic CNN architecture, has several unique aspects that make it well-suited for image classification tasks:

1. Weight sharing: LeNet utilizes weight sharing in its convolutional layers. The same set of weights is applied to different spatial locations across the input. This sharing of parameters enables the network to extract and recognize similar features throughout the image. It helps reduce the total number of parameters and allows LeNet to efficiently capture local patterns.
2. Activation function: LeNet employs the sigmoid activation function in its fully connected layers. The sigmoid function squashes the output of each neuron into a range between 0 and 1. This non-linearity introduces non-linear transformations and allows the network to model complex relationships between features.
3. Convolution and pooling operations: LeNet incorporates convolutional layers for spatial feature extraction. Convolutional layers apply filters to the input, capturing local patterns and creating feature maps. Pooling operations, specifically max pooling in LeNet, downsample the feature maps via selecting the maximum value in each pooling region which reduces the spatial dimensions of the features.
4. Architectural simplicity: LeNet architecture consists of alternating convolutional and pooling layers followed by fully connected layers. Overall, LeNet’s unique features of weight sharing, sigmoid activation, and the combination of convolutional and pooling operations made it a pioneering architecture for image classification tasks and achieving higher performance.

## 5. Modified LeNet Architecture

The modified LeNet architecture is trained on a large-scale image classification dataset such as ImageNet, which contains over 1 million images with 1000 classes. The training process includes data augmentation techniques such as random clipping, horizontal flips, and dithering to improve model robustness and prevent overfitting. After training the individual models, ensembling techniques combine their predictions. Specifically, a simple averaging method is used where the outputs of the unique models are averaged to obtain the final prediction. The classifier uses more advanced ensembling techniques, such as stacking and boosting, which can further improve accuracy.

**Figure 4:**
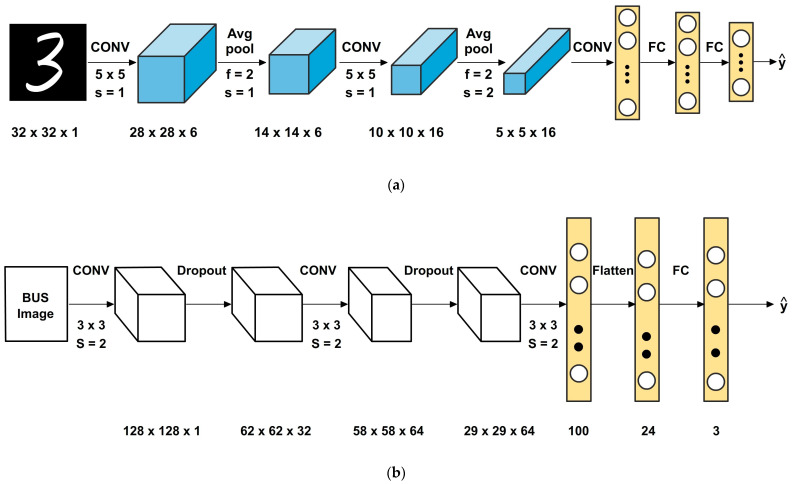
(**a**) Classical LeNet architecture. (**b**) Modified LeNet architecture.

The traditional LeNet-5 architecture is shown in Figure 4a which consists of the convolutional layer with six filters of size 5 × 5, a stride of 1, no padding, and Sigmoid as an activation function. A 5 × 5 convolutional layer with stride 1 and no padding has a receptive field of 5 × 5, which can be too large for specific image classification tasks. A large receptive field can cause the model to lose some spatial information and fine-grained details in the input image. The 5 × 5 convolutional layers typically have a higher computational cost than 3 × 3 convolutional layers, especially when the number of input channels is large. This can make the training and inference of the model slower and more computationally expensive.

In this proposed modified LeNet model (presented in Figure 4b), the 5 × 5 layers are replaced using two 3 × 3 layers for better feature extraction. The intuition behind this modification is that stacking two 3 × 3 convolutional layers one after the other allows the model to learn more complex and abstract features, while keeping the same receptive field as a single 5 × 5 convolutional layer. This is because a 3 × 3 convolutional layer with stride 2 and no padding has a receptive field of 3 × 3 and stacking two of these layers in sequence results in a receptive field of 5 × 5. Further, using two 3 × 3 convolutional layers can result in more significant nonlinearity, improving the model’s ability to learn complex and non-linear relationships between the input image and the target labels. The batch normalization is applied after each convolutional layer. It normalizes the activations, stabilizing and accelerating the training process.

The original pooling layers are replaced with convolutional layers having a stride of 2. This achieves downsampling while enabling the network to learn more intricate representations. The sigmoid activation function of the traditional LeNet is replaced with the Rectified Linear Unit (ReLU) [51] activation function. ReLU mitigates the vanishing gradient problem and improves training convergence. Dropout regularization is introduced after specific layers which are shown in Figure 4b. It randomly deactivates a fraction of input units during training, helping prevent overfitting and enhancing generalization. The modified architecture is made deeper via adding additional convolutional and/or fully connected layers after the initial layers. This increases the network’s capacity to learn complex patterns. The modified architecture involves ensembling multiple CNN models. Ensembling combines multiple models to improve the overall performance and robustness of predictions. Instead of relying on a single model, ensembling leverages the wisdom of crowds via aggregating the predictions from multiple models to make a final prediction. Modified LeNet models would be creating an ensemble, trained on different subsets of the training data or with different initialization. The final prediction of the ensemble would be determined through soft voting or averaging of the predictions from the individual models.

### 5.1. Batch Normalization

Batch normalization [17] is a technique that the LeNet model can use to improve the performance of neural networks, including various image classification tasks that include breast cancer detection using ultrasound images. Here are some advantages of using batch normalization in LeNet for breast cancer detection. Batch normalization can help accelerate the confluence of the training process via reducing the internal covariate shift, which refers to the change in the distribution of the input to a layer due to the changing weights in the previous layers and improve convergence. Batch normalization [17] can reduce internal variability via normalizing data at each layer and help stabilize gradients during recovery quickly. The advantages are regularization, improved accuracy, and greater flexibility. LeNet with batch normalization for breast cancer detection using ultrasound images can improve convergence, regularization, accuracy, and flexibility, all of which can contribute to better performance on this task.

### 5.2. Replacing the Pooling Layers

The rationale behind replacing the pooling layers with convolutional layers of stride 2 in the LeNet architecture is to improve information retention, enhance feature extraction, and potentially enhance the model’s performance [51]. The reasons behind this modification lie in the potential limitations of pooling operations. Pooling operations can discard some information from the feature maps, especially if the pooling kernel size is large or the stride is high. By replacing the pooling layers with convolutional layers of stride 2, more information can be retained in the feature maps. This can be beneficial for preserving important details and preventing the loss of valuable information during the downsampling process.

Convolutional layers can learn more complex and abstract features than pooling layers, especially when they have more parameters or are deeper. The model can extract more relevant features from the input images utilizing convolutional layers instead of pooling layers, ultimately improving the accuracy of the classification task. Another advantage of this modification is the potential reduction in computational cost. Pooling operations can be computationally expensive, especially with large kernel sizes or high strides. The computational burden can be reduced via replacing the pooling layers with convolutional layers of stride 2, making the model faster to train and evaluate. Overall, replacing the pooling layers with convolutional layers of stride 2 enhances information retention, feature extraction, model performance, and computational efficiency within the LeNet architecture.

### 5.3. ReLU Activation

The activation of each layer in a neural network controls the activation of the preceding layer and contributes to the nonlinearity of the overall network. The proposed architecture introduces a mesh consisting of five convolutional stack normalization groups. Replacing the sigmoid activation function with the ReLU activation function effectively alters the LeNet architecture for diagnosing cancer using ultrasound images. This modification brings several benefits, including improved performance, faster computation, increased learning capacity, enhanced abstraction, and mitigating the vanishing gradient problem. The vanishing gradient problem arises when the gradient of the activation function becomes too small, impeding the adjustment of weights during training. ReLUs are less prone to this issue than sigmoid processing, enabling the network to effectively learn the characteristics and patterns within ultrasound images [51]. This modification aims to enhance accuracy, improve comprehension, and increase specificity.

### 5.4. Dropout

**Figure 5:**
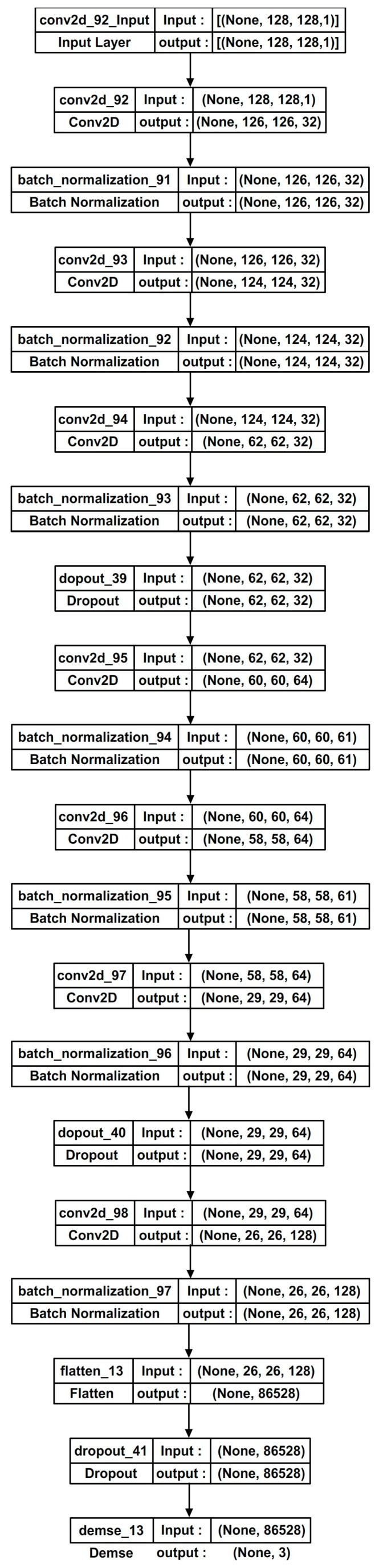
Layers in the modified LeNet model.

Adding dropouts to the LeNet architecture for breast cancer detection can be useful. Dropout is a regularization technique that randomly drops out (sets to zero) some of the activations in the network during training [22,51]. The percentage of dropouts used is 40% (0.4) for all the dropout layers (Dropout(0.4)). Four dropout layers exist: two after the convolutional layers and two before the final dense layer. Dropout can help prevent overfitting of the model to the training data via reduction of the co-adaptation of neurons. This improves the model’s ability to expand to new, unseen data, which is important for accurate breast cancer detection and performing robust learning [51]. By randomly dropping out activations during training, the network is forced to rely on a wider range of features and patterns in the input data. This can improve the model’s robustness to input data variations, such as differences in patient demographics or imaging conditions. Dropout can help reduce the model’s sensitivity to the network’s initial conditions (i.e., the weights and biases). This increases the reproducibility of the results and reduces the risk of the model getting stuck at the local minimum during training. By lowering the co-adaptation of neurons and forcing the network to rely on a broader range of features, dropout can make the training process more efficient and less prone to becoming stuck in local minima [33]. Figure 5 shows the various layers in the modified LeNet model.

## 6. Materials and Methods

### 6.1. Dataset Description

The “Breast Ultrasound Images Dataset” is available at Kaggle [50]. It contains 971 breast ultrasound images in JPEG format and their corresponding labels (0, 1, and 2) indicating a resolution of 500 × 500 pixels. The images are categorized into three classes, which are normal, benign, and malignant.

### 6.2. Software Details

Google Colab, a cloud-based Jupyter notebook environment was used for conducting our experiments. Google Colab was connected to Google Drive to access the image sources. The presence of GPUs in Google Colab, specifically configured with type T4, greatly expedited the model training process. Deep learning models are built using the Keras 2.6.0 library, which is a user-friendly neural networks API that operates on top of TensorFlow 2.7.0, a robust open-source machine learning framework. The seamless integration of Keras and TensorFlow with Google Colab allowed the effortlessly import of the necessary libraries and executed code within a collaborative environment. This integration not only facilitated the development of the models but also maximized the utilization of GPU resources. Python 3.10.11 is used for the implementation.

## 7. Performance Metrics

To demonstrate the performance of the proposed model, breast ultrasound images are used. Our model was trained for 40 epochs. The accuracy of the breast image recognition rate is 89.91%. The various performance evaluation metrics are discussed below.

### 7.1. Classification Accuracy

Classification accuracy is a metric used to evaluate the performance of a classifier model during the training phase. It measures the proportion of correctly classified instances, both positive and negative, out of the total instances in the training set. It is usually defined as the percentage of samples excluded from the training process. Based on this, the training accuracy is represented in Equation (1):

Accuracy = TP + TN/(TP + TN + FP + FN)(1)

where TP represents true positives; TN represents true negatives; FP represents false positives; and FN represents false negatives. TP is defined as the cases where the model correctly predicts the positive class. TN is defined as the cases where the model correctly predicts the negative class. FP is defined as the cases where the model incorrectly predicts the positive class. FN is defined as the cases where the model incorrectly predicts the negative class. The training accuracy metric provides a general overview of how well the model can classify instances correctly during training. Training accuracy metrics provide insights into different aspects of the model’s behavior, such as its ability to handle imbalanced classes or its performance in specific classes.

Furthermore, it is important to split the data into separate training and testing sets to accurately evaluate the model’s performance on unseen data. This helps in identifying and mitigating issues such as overfitting, where the model becomes overly specialized to the training data and fails to generalize well. In conclusion, while training accuracy is a valuable metric to assess the model’s performance during training, it should not be considered in isolation. It is crucial to analyze various evaluation metrics, use separate validation and testing sets, and consider the model’s performance on unseen data to gain a more comprehensive understanding of its effectiveness.

### 7.2. Validation Loss

Validation loss is a valuable metric for assessing model performance on unseen data. It is crucial to guide model selection, implement early stopping, and optimize hyperparameters to improve this model’s generalization and predictive capabilities. The validation loss is calculated via feeding the dataset (a subset of the total dataset not used for training) through the trained model and computing the loss on this dataset and is presented in Equation (2):

L = −(1/N) × ∑(y × log(y_pred) + (1 − y) × log(1 − y_pred))(2)

where N is the number of samples in the validation dataset; y is the true label (either 0 or 1); and y_pred is the predicted probability of the positive class (i.e., the class with label 1). Results are obtained from all N samples in valid data. The total validation loss is the average of all sample losses is presented in Equation (3):

Val_loss = (1/N) × ∑(L)(3)

### 7.3. Precision

Precision measures the proportion of correctly predicted positive samples out of all the samples predicted as positive and is presented in Equation (4):

Precision = TP/(TP + FP)(4)

### 7.4. Recall

Recall (also known as sensitivity or true positive rate) measures the proportion of correctly predicted positive samples out of all the actual positive samples and is presented in Equation (5):

Recall = TP/(TP + FN)(5)

### 7.5. F1 Score

The F1 score is the harmonic mean of precision and recall, providing a balanced measure of model performance which is presented in Equation (6):

F1 Score = 2 × (Precision × Recall)/(Precision + Recall)(6)

## 8. Results and Discussion

**Figure 6:**
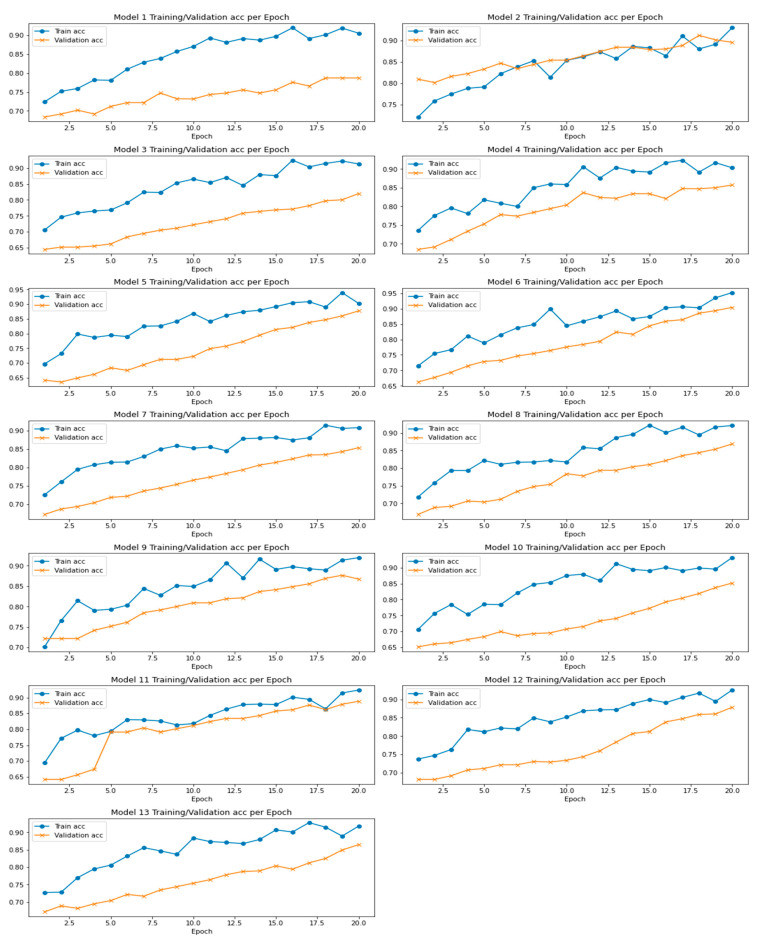
Training and validation Accuracy per epoch for ensemble of 13 LeNet Models in BUS Dataset.

The proposed ensembled LeNet CNN outperforms the original LeNet architecture and performs comparably to other state-of-the-art CNN architectures. According to our experimental findings, the ensembled LeNet CNN models considerably increased the dataset’s breast image recognition accuracy, especially when the individual models have different strengths and weaknesses. Experimental evaluation of an ensembled LeNet CNN for breast cancer analytics would involve training multiple LeNet models on a breast cancer dataset and then combining their predictions using some ensemble method. It is worth noting that the choice of ensemble method and hyperparameters can significantly impact the performance of the ensembled LeNet CNN. Therefore, different ways and settings should be tested to find the best combination for the given data and the problem to be solved. Training/validation accuracy per epoch for all the 13 models is shown in Figure 6.

Experimental evaluation of ensembled LeNet CNN for breast cancer analytics is performed as follows:

1. The first step is to preprocess the BUS dataset to ensure it is in the appropriate format for training and testing models. This will include resizing the image, normalizing the pixel values, and dividing the data into training, testing sets, and validation sets.
2. Train multiple LeNet models with different initialization or hyperparameter settings on the training set. This can be achieved using the TensorFlow deep learning framework.
3. Combine the predictions of the individual LeNet models using soft voting.
4. Hyperparameter tuning optimizes the model’s performance via finding optimal values for different hyperparameters. The number of filters in convolutional layers affects model complexity. Kernel size captures spatial patterns. Adjusting dropout rate prevents overfitting. Learning rate in Adam optimizer controls gradient descent step size. Random search samples hyperparameters randomly within a predefined range and provides computational efficiency for exploring the hyperparameter space.
5. Evaluate the performance of the LeNet CNN cluster via calculating parameters such as accuracy, precision, recall, and F1 score. Compare the overall performance of a LeNet model and other high-end models.

### 8.1. Modified LeNet’s Performance Scores

From Table 1, it is understood that the model achieved a good balance between precision and recall, considering both false positives and false negatives. A higher precision score indicates that the model has a low false positive rate, meaning it correctly identifies positive instances without wrongly classifying negative instances. A higher recall score suggests that the model has a low false negative rate, meaning it successfully identifies most positive instances without missing many. The F1 score suggests that the model’s predictions have a good balance between correctly identifying positive instances and minimizing false positives and false negatives. Ensembling, ReLU activation function, and 40% dropout that are incorporated in LeNet have contributed to these performance scores.

The ensemble method combines multiple models to improve overall prediction accuracy, while the LeNet architecture is specifically designed for BUS image recognition tasks. Dropout regularization can help prevent overfitting via randomly dropping out a fraction of the units during training. The ReLU activation function is commonly used in deep learning models as it helps introduce non-linearity and can improve the model’s ability to learn complex patterns. Overall, the modified LeNet model appears to perform well, with high accuracy, precision, recall, and F1 score values, indicating an effective classification performance.

### 8.2. Experimental Evaluation

With ensembled LeNet CNN, breast cancer classification is successfully completed. 540 images have been used for training, 180 images for testing, and 180 images for validation and these images (40 nos. only) are represented in Table 2 (indexed with Image ID) which are fed into each LeNet CNN model which predicted the output (0 → normal, 1 → benign, and 2 → malignant) and finally the majority of the outputs of the thirteen ensemble models (shown in Table 1) is declared as a result. For example, in Image ID 1, the first LeNet CNN model predicted 0 which means normal, while the rest of the ensemble models predicted 1, which means benign. Since most models indicated it as 1, the result is also 1. The same procedure is followed for all 40 imageids. The average runtime taken for each iteration is the 1750s which is much better than the average runtime of classical LeNet (2120s).

#### 8.2.1. Modified LeNet Model’s Predictions Using BUS Dataset

During the training process, early stopping was triggered at the 13th iteration, after 27 epochs. At this point, the proposed model demonstrated higher accuracy than the traditional neural network and LeNet models. This indicates that the proposed model outperformed the other models significantly, showcasing superior performance and its effectiveness. The use of modified LeNet in model construction allows for early stopping. The classifier has made predictions on medical images, where the labels indicate whether the image shows normal, benign, or malignant tissue. Table 2 shows the soft voting-based ensemble learning method that combines the predictions of multiple individual classifiers to make a final prediction. Each individual classifier gives a probability distribution over the possible classes, and the soft voting classifier takes the average of these probabilities to make the final prediction.

**Figure 7:**
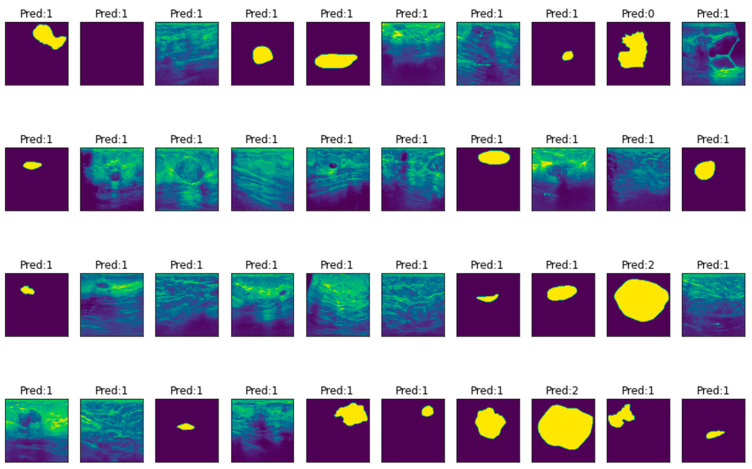
Predictions for BUS dataset obtained using modified LeNet showing normal, malignant, and benign classifications.

Early stopping is commonly used in machine learning to prevent overfitting and find the optimal training point. It involves monitoring a chosen metric, such as validation loss or accuracy, during training. Training is stopped early to prevent overfitting and retain the best-performing model when the metric stops improving or consistently starts to deteriorate. In Table 3 of the ensembled runs, when early stopping (due to hyperparameter tuning) is triggered during the training process, the model weights from the iterations that achieved the best performance are typically saved. This saved model corresponds to the best-performing iteration (e.g., the 13th iteration) and is then utilized for inference or further evaluation on the test set. Figure 7 shows the predicted images for normal, benign, and malignant. From the empirical results, it is observed that the changes performed in the modified LeNet model reduced the computational time and thus enhanced the classification performance (compared to classical LeNet).

#### 8.2.2. Modified LeNet Model’s Performance Analysis

The classifier’s predictions for each Image ID is presented in Table 2. Values 1, 2 and 3 in the “won” in Table 2 represent normal tissue, malignant tissue, and benign tissue, respectively. The classifier has apparently predicted that most of the images are either normal or benign, with only a few images (IDs 6, 28, and 36) being predicted as malignant. Experimental results are shown in Figure 7. The results are very much identical to the clinician’s results and from Figure 7, malignant, benign, and normal classes obtained based on the BUS dataset are clearly depicted. This is possible because of the model’s ability to obtain much better results from the modifications that are made in modified LeNet. From this, it can be claimed that the proposed modified LeNet model “WON” in the agreement between the model and clinicians. This means that the model’s predictions align perfectly with the expert judgment of the clinician. Overall, the classifier is useful in classifying breast tissues into malignant (pred:2, in Figure 7), benign (pred:1, in Figure 7), and normal (pred:0, in Figure 7). When mapped with the trained data, the predicted class is accurately matching.

In Table 4, the performance metrics (accuracy, validation accuracy, loss, and validation loss) of various models on the BUS dataset are observed [47,48]. The performance metrics provide insights into how well each model performs in terms of classification accuracy and the amount of error or loss during training and validation. The accuracy metric indicates the proportion of correctly classified samples. From Table 4, the modified LeNet model achieves the highest accuracy of 0.8991, indicating that it correctly classifies (approximately) 89.91% of the samples. It outperforms all other models in terms of accuracy. On the other hand, the “SCAN” model has the lowest accuracy of 0.8056. The validation accuracy metric measures the model’s performance on unseen validation data. Like accuracy, the “Modified LeNet” model achieves the highest validation accuracy of 0.7652, indicating good generalization ability. The “U-Net” model has the lowest validation accuracy of 0.7003.

The loss metric represents the error or discrepancy between predicted and actual values during training. Lower loss values indicate better model performance. The “Dense U-Net” model has the lowest training loss of 1.1125, indicating a better fit to the training data. Conversely, the “U-Net” model has the highest loss of 1.7426. Dense U-Net has the lowest value for validation loss which is 1.4698 and next to it, modified LeNet has 1.7707 suggesting better generalization on unseen validation data. In summary, the “Modified LeNet” model outperforms other models on the BUS dataset based on the provided metrics It achieves the highest accuracy and validation accuracy while maintaining relatively low loss values. This suggests that the “Modified LeNet” model might be the most effective among the models considered for the BUS dataset.

## 9. Conclusions

Deep learning models, like CNNs, have shown promising results in breast cancer prediction using medical images. However, a single CNN model may not consistently achieve the desired accuracy or robustness. Batch learning is a technique that combines predictions of multiple models to improve performance. Evaluating the ensembled LeNet CNN for breast cancer prediction combines multiple LeNet models, each trained on the same dataset with different initialization or hyperparameter settings. The predictions of the individual models are combined using soft voting techniques. LeNet CNN cluster includes comparing its performance with a LeNet model and other cutting-edge models using metrics such as accuracy, precision, recall, and F1 score. The choice of mixing operation and hyperparameters can affect the performance of the model. The empirical results show the effectiveness of ensembled CNN models for breast cancer prediction. In one study, an ensembled CNN achieved an accuracy of 85.57% on a breast cancer dataset, outperforming a single CNN model and other state-of-the-art models. The thirteen LeNet CNN models ensembled had an accuracy of 89.91%. The use of ensembling allows for the combination of multiple models to mitigate the drawbacks of individual models, such as overfitting and bias. The proposed work shows that ensembling LeNet CNN models can significantly increase the accuracy of image classification tasks and can help reach more excellent accuracy rates. In future, a clustering LeNet CNNs is a promising method to improve the accuracy and robustness of breast cancer prediction using clinical images, and their effectiveness should be further investigated.

## Figures and Tables

**Table 1:** Precision, recall and F1 score of modified LeNet.

Method	Precision	Recall	F1 Score
Modified LeNet	0.85	0.92	0.88

**Table 2:** Predictions (for 40 images) made using the proposed model using soft voting approach.

Image ID	Won	Image ID	Won	Image ID	Won	Image ID	Won
	1	11	1	21	1	31	1
2	1	12	1	22	1	32	1
3	1	13	1	23	1	33	1
4	1	14	1	24	1	34	1
5	1	15	1	25	1	35	1
6	1	16	1	26	1	36	2
7	1	17	1	27	1	37	1
8	1	18	1	28	2	38	1
9	0	19	0	29	0	39	0
10	1	20	1	30	1	40	1

**Table 3:** Ensembled LeNet iterations to find the best performing model.

Models	Accuracy	Val_Accuracy	Loss	Val_Loss
Net1	0.8493	0.7394	1.4750	2.1780
Net2	0.8427	0.85906	1.5121	2.1867
Net3	0.8402	0.7258	1.4423	1.9361
Net4	0.8557	0.7943	1.3765	2.1965
Net5	0.8427	0.7459	1.4236	1.9475
Net6	0.8522	0.7856	1.2977	1.5963
Net7	0.8470	0.7681	1.3175	2.2156
Net8	0.8469	0.7698	1.4092	2.3700
Net9	0.8459	0.7322	1.3739	2.1124
Net10	0.8469	0.7469	0.4733	0.4431
Net11	0.8396	0.7983	1.5013	1.7907
**Net12**	**0.8991**	**0.7652**	**1.3770**	**1.7707**
Net13	0.8516	0.7591	1.4053	1.8033

**Table 4:** Comparison of proposed model with pre-trained models on the BUS dataset.

Models	Accuracy	Val_Accuracy	Training_Loss	Val_Loss
AlexNet	0.8725	0.7325	1.6250	2.0125
SegNet	0.8127	0.7426	1.3232	2.3625
U-Net	0.8619	0.7003	1.7426	1.9981
CE-Net	0.8352	0.6982	1.3526	2.2635
SCAN	0.8056	0.7169	1.3667	1.9478
Dense U-Net	0.8102	0.7456	1.1125	1.4698
LeNet	0.8506	0.7223	1.3225	2.2215
**Modified LeNet**	**0.8991**	**0.7652**	**1.3770**	**1.7707**

## Data Availability

Synthetic datasets [49,50] are used to conduct experiments.
